# Hyperintense Acute Reperfusion Marker on FLAIR in Posterior Circulation Infarction

**DOI:** 10.1371/journal.pone.0157738

**Published:** 2016-06-21

**Authors:** Alex Förster, Holger Wenz, Johannes Böhme, Mansour Al-Zghloul, Christoph Groden

**Affiliations:** Department of Neuroradiology, Universitätsmedizin Mannheim, University of Heidelberg, Mannheim, Germany; University Medical Center (UMC) Utrecht, NETHERLANDS

## Abstract

**Purpose:**

In the present study, we aimed to investigate the frequency of blood brain barrier injury in posterior circulation infarction as demonstrated by the hyperintense acute reperfusion marker (HARM) on fluid attenuated inversion recovery images (FLAIR).

**Methods:**

From a MRI report database we identified patients with posterior circulation infarction who underwent MRI, including perfusion-weighted images (PWI), within 12 hours after onset and follow-up MRI within 24 hours and analyzed diffusion-weighted images (DWI), PWI, FLAIR, and MR angiography (MRA). On FLAIR images, the presence of HARM was noted by using pre-specified criteria (focal enhancement in the subarachnoid space and/or the ventricles).

**Results:**

Overall 16 patients (median age of patients 68.5 (IQR 55.5–82.75) years) with posterior circulation infarction were included. Of these, 13 (81.3%) demonstrated PCA occlusion, and 3 (18.7%) patients BA occlusion on MRA. Initial DWI demonstrated ischemic lesions in the thalamus (68.8%), splenium (18.8%), hippocampus (75%), occipital lobe (81.3%), mesencephalon (18.8%), pons (18.8%), and cerebellum (50%). On follow-up MRA recanalization was noted in 10 (62.5%) patients. On follow-up FLAIR images, HARM was observed in 8 (50%) patients. In all of these, HARM was detected remote from the acute ischemic lesion. HARM was more frequently observed in patients with vessel recanalization (p = 0.04), minor infarction growth (p = 0.01), and smaller ischemic lesions on follow-up DWI (p = 0.05).

**Conclusions:**

HARM is a frequent finding in posterior circulation infarction and associated with vessel recanalization, minor infarction growth as well as smaller infarction volumes in the course. Neuroradiologists should be cognizant of the fact that HARM may be present on short interval follow-up FLAIR images in patients with acute ischemic infarction who initially underwent MRI and received intravenous gadolinium-based contrast agents.

## Introduction

Acute ischemic stroke in the vertebrobasilar or posterior circulation accounts for up to 25% of all ischemic strokes.[1] Regarding the supplying vessels (the vertebral arteries (VA) merge and form the single midline basilar artery), the higher frequency of congenital vessel variations (e.g. hypoplasia of the VA), and the large variety of supplied anatomical structures (neocortex, allocortex, thalamus, brainstem, and cerebellum) the anatomy of the posterior circulation differs significantly from the anterior circulation.[2] Furthermore, differences in stroke etiologies[3] and complications in the clinical course, such as secondary intracerebral hemorrhage have been reported.[4] The latter might be explained by differences in blood brain barrier impairment as was suggested recently.[5] For the evaluation of blood brain barrier impairment in acute ischemic stroke qualitative and quantitative MRI techniques have been investigated and reported such as permeability images derived from dynamic susceptibility contrast (DSC) perfusion-weighted imaging (PWI)[5], and dynamic contrast enhanced (DCE) PWI.[6] Another promising MRI based approach is the detection of the hyperintense acute reperfusion marker (HARM) on fluid attenuated inversion recovery (FLAIR) images,[7] which is caused by blood-brain barrier disruption following acute recanalization and reperfusion and consecutive delayed gadolinium contrast enhancement in the subarachnoid space on fluid attenuated inversion recovery (FLAIR) images[8]. However, so far no dedicated studies focusing on HARM in posterior circulation infarction have been published. Therefore, we aimed to investigate the frequency of HARM in posterior circulation infarction and its relation to recanalization and infarction growth.

## Material and Methods

### Patients

From a prospectively maintained MRI report database (Syngo Data Manager–SDM), we identified patients with acute ischemic stroke due to occlusion of the PCA or BA who underwent a standard stroke MRI protocol including PWI (2005–2016). Of these, only those patients were included who had the first MRI within 12 hours after onset as well as follow-up MRI within 24 hours after the first MRI. The demographic details, clinical presentation, and acute treatment were abstracted from the case records. Patient records, medical images, and other data were not anonymized before access for this study. This study has been approved by the local institutional review board (Medizinische Ethikkommission II der Medizinischen Fakultät Mannheim) and has therefore been performed in accordance with the ethical standards laid down in the 1964 Declaration of Helsinki and its later amendments. Patient consent was waived for this analysis by the local institutional review board due to its retrospective nature.

### MRI studies

Magnetic resonance imaging was performed on a 1.5-T MR system (Magnetom Sonata or Avanto, Siemens Medical Systems, Erlangen, Germany). A standardized protocol was used in all patients including (1) transverse, coronal and sagittal localizing sequences followed by transverse oblique contiguous images aligned with the inferior borders of the corpus callosum (applied on sequences 2 to 6); (2) T1-weighted images; (3) T2-weighted images; (4) diffusion-weighted images (DWI); (5) FLAIR images (field of view 205 mm × 230 mm, matrix 448 mm × 304 mm, number of slices 24, slice thickness 5 mm, TR 8500/9000 ms, TE 115/89 ms, TI: 2400/2500 ms for Magnetom Sonata/Avanto); (5) T2*-weighted images; (6) PWI following the first pass of contrast bolus (gadoteric acid (Dotarem, Guerbet, Aulnay-sous-Bois, France) with a dose of 0.1 mmol/kg of body weight at a rate of 4 ml/sec) through the brain; and (7) a 3D time-of-flight MR angiography (MRA). Follow-up MRI was performed using the same protocol except for PWI which was performed in only 2 patients.

### Postprocessing of perfusion maps

The postprocessing of the perfusion-weighted raw images was performed by a specific software, Signal Processing In NMR (SPIN, The MRI Institute for Biomedial Research, Detroit, USA) [9]. Deconvolution with singular value decomposition (SVD) was used to create quantitative maps of CBF, and CBV. The position of the arterial input function (AIF) was automatically determined by using the maximum concentration (Cmax), TTP and first moment MTT (fMTT). The concentration-time curve for arteries has short fMTT, short TTP and high Cmax. Twenty voxels, which best fitted these properties were selected. Then the concentration-time curves of these voxels were averaged, smoothed and truncated to avoid the second pass of the tracer.

### MRI analysis

Acute ischemic lesions were noted on DWI, the topography determined according to the maps by Tatu et al. [10,11]. and categorized in (1) PCA, (2) mesencephalon, (3) pons, (4) medulla oblongata, and (5) cerebellum. In the PCA territory acute ischemic lesions were further categorized in (1) thalamus, (2) splenium, (3) hippocampus, (4) occipital lobe, and (5) mesencephalon. Ischemic lesion size was measured on DWI by manually delineated ROI, summation of these areas in cm^2^ on each section and multiplication with the slice thickness (plus interslice gap), to determine the volume in cm^3^ by use of OsiriX (Pixmeo SARL, Bernex, Switzerland), a multidimensional image navigation and display software [12]. Lesion growth as well as reversal were defined as the difference between the infarction volume on follow-up DWI images and the initial DWI lesion volume. Additionally, vessels of the circle of Willis were determined on the 3D TOF stack by use of OsiriX. MR angiography was classified as normal, stenosis, and vessel occlusion. On follow-up FLAIR images, the presence or absence of HARM was noted by using pre-specified criteria. Criteria for HARM were focal enhancement on FLAIR images in the subarachnoid space and/or the ventricles [13]. In unilateral PCA occlusion, the perfusion maps were also quantitatively assessed by use of SPIN: a region of interest (ROI) covering the hypoperfused area was placed on the generated maps (CBF, CBV) and mirrored to the contralateral unaffected hemisphere. Finally, ratios between the physiological estimates (CBF, CBV) of the lesion and of the contralateral mirror ROI were determined.

### Statistical analysis

All statistical analyses were performed using Statistical Product and Service Solutions (SPSS) statistics for Windows (Release 17.0; SPSS, Chicago, IL, USA). Descriptive data was analyzed by use of Chi-square tests and the Mann-Whitney U Test as appropriate. Comparison between patients with or without HARM was performed using Chi-square tests and the Mann-Whitney U Test as appropriate. Comparison of lesion size on DWI and PWI was performed using the Mann-Whitney U Test or the Wilcoxon Test as appropriate. All statistics was performed with a 0.05 level of significance.

## Results

### Baseline characteristics and clinical presentation

Overall, we identified 61 patients with PCA or BA occlusion. In the final analysis, 16 (26.2%) patients were included who met the pre-specified inclusion criteria. For details see S1 Table. Of these, 13 (81.3%) patients had a PCA occlusion and 3 (18.7%) patients a BA occlusion. The median age of patients was 68.5 (IQR 55.5–80.75) years; 9 (56.3%) patients were male, 7 (43.8%) female. Comorbidities and cerebrovascular risk factors included arterial hypertension (81.3%), hyperlipidemia (50%), smoking habit (25%), transient ischemic attack/stroke (18.8%), diabetes mellitus (12.5%), coronary heart disease (12.5%), and chronic kidney disease (12.5%). Clinical symptoms comprised hemiparesis (75%), hemihypaesthesia (50%), dysarthria (43.8%), hemianopia (43.8%), and aphasia (18.8%), while other symptoms like altered vigilance, vertigo, oculomotor dysfunction, ataxia, and hemineglect were only observed occasionally. Intravenous thrombolysis with rtPA was performed in 12 (75%) patients.

#### MRI analysis

Initial MRI was performed within a median time of 111.5 (IQR 84.75–356.25) minutes after onset of symptoms. DWI demonstrated an acute ischemic infarction in the thalamus in 11 (68.8%), in the splenium in 3 (18.8%), in the hippocampus in 12 (75%), in the occipital lobe in 13 (81.3%), in the mesencephalon in 3 (18.8%), in the pons in 3 (18.8%), and in the cerebellum in 8 (50%) patients. Additional lesions outside the posterior circulation were noted in the anterior cerebral artery territory in 1 (6.3%) patient. Ischemic lesions on DWI had a median volume of 3.2 (IQR 0.70–5.05) cm^3^. In all patients, PWI showed an area of hypoperfusion that extended beyond the observed DWI lesion. The hypoperfused areas had a median volume of 30.85 (IQR 23.53–37.5) cm^3^ while the corresponding median DWI lesion size was significantly smaller (p<0.001). In the hypoperfused areas the median CBF and CBV ratios were 0.71 (IQR 0.61–0.97) and 0.91 (IQR 0.74–1.10) respectively.

Follow-up MRI was performed within a median time of 16 (7.2–20.75) hours after the inital MRI. On follow-up DWI, the ischemic lesions had a significantly larger median volume of 5.0 (IQR 1.85–17.9) cm^3^ (p = 0.003). A partial or complete recanalization was observed in 10 (62.5%) patients.

**Fig 1:**
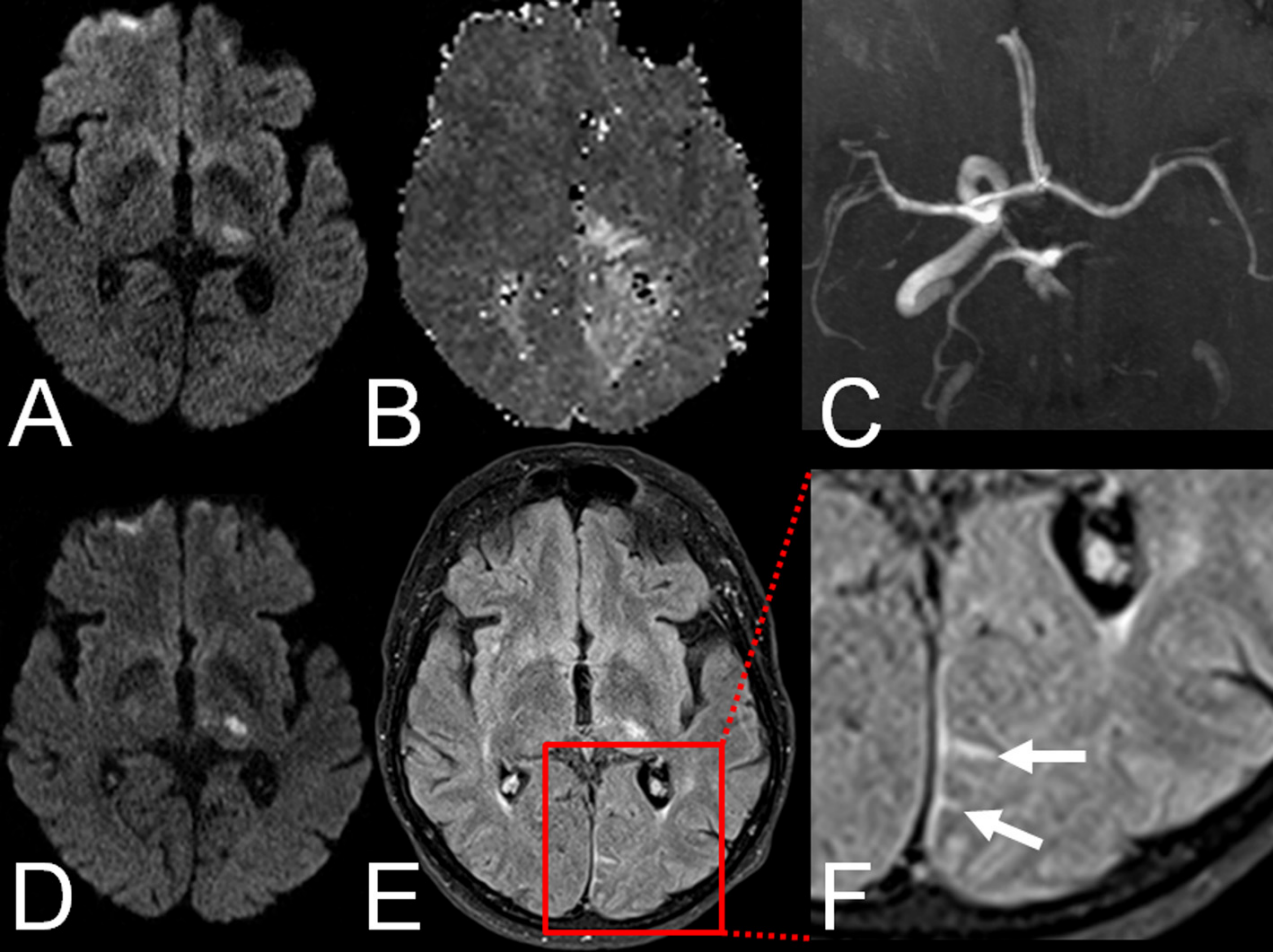
Example of HARM in a 67-year old patient with left posterior cerebral artery (PCA) infarction: A. Acute ischemic lesion in the left thalamus on DWI. B. Hypoperfusion in the left PCA territory on PWI. C. Proximal occlusion of the left PCA on TOF-MRA. D. Acute ischemic lesion in the left thalamus on follow-up DWI. E. HARM in the left PCA territory on FLAIR images. E. Magnification (1.5x) of HARM on FLAIR images (arrows).

On follow-up FLAIR images, HARM was observed in 8 (50.0%) patients. In 1 (12.5%) patient with initial BA occlusion HARM was found in the ambient cistern; in the remaining 7 (87.5%) patients with initial PCA occlusion in occipital sulci. For an example see Fig 1. Notably, in all of these cases HARM was detected in the same vascular territory as the stroke but remote from the acute ischemic lesions. The presence of blood as the cause of the increased sulcal signal on FLAIR images could be ruled out on T2*-weighted images which never demonstrated corresponding sulcal hypointensities. HARM was more frequently observed in patients with vessel recanalization (p = 0.04). Furthermore, patients with detection of HARM had only minor infarction growth (p = 0.01) as well as smaller ischemic lesions on follow-up MRI (p = 0.05). For a detailed comparison see Table 1.

**Table 1:** Demographic characteristics, ischemic lesion size on initial and follow-up DWI, as well as perfusion deficit size on initial PWI in patients with and without HARM.

median (IQR),	HARM,	No HARM	OR; 95%CI	p
unless noted	n = 8	n = 8		
**Age, years**	67.5 (63.75–73)	73 (41–83.5)		0.72
**Male sex (n, %)**	5 (62.5%)	4 (50%)	1.67; 0.23–12.22	0.61
**CKD**	1 (12.5%)	1 (12.5%)	1.00; 0.05–19.36	1.00
**Time between onset**	104 (85–117)	336 (77–360)		0.46
**and MRI, minutes**				
**Time between**	10.5 (5.25–17.75)	19.5 (15.5–23.5)		**0.03**
**MRI 1/2, hours**				
**MRI 1**				
**DWI lesion size, cm**^**3**^	3.55 (0.55–4.28)	2.05 (0.75–10.45)		0.72
**PWI lesion size, cm**^**3**^	30.85 (13.5–41.25)	30.9 (25–37.5)		0.72
**rCBF**	0.65 (0.58–0.97)	0.85 (0.57–0.97)		0.63
**rCBV**	0.89 (0.67–1.05)	1.02 (0.70–1.17)		0.37
**MRI 2**				
**DWI lesion size, cm**^**3**^	3.3 (1.03–8.23)	16.35 (4.4–35.18)		**0.05**
**Recanalization**	7 (87.5%)	3 (37.5%)	11.67; 0.92–147.56	**0.04**
**Infarction growth, cm**^**3**^	0.15 (-0.15–3.55)	7.6 (3.35–31.35)		**0.01**

Legend: HARM = hyperintense acute reperfusion marker, CKD = chronic kidney disease, DWI = diffusion-weighted imaging, PWI = perfusion-weighted imaging

## Discussion

Acute ischemic infarction in the posterior circulation varies significantly from acute ischemic infarction in the anterior circulation in different ways such as minor incidence, clinical presentation, as well as anatomical and vascular characteristics. Whether changes in blood brain barrier permeability in acute ischemic stroke differ between anterior and posterior circulation remains unclear [5]. In 2004, HARM has been described in acute ischemic stroke for the first time [7,14]. The phenomenon is caused by blood-brain barrier disruption following acute recanalization, reperfusion and consecutive delayed gadolinium contrast enhancement in the subarachnoid space on FLAIR images [8]. The present study describes three novel and essential findings regarding HARM in posterior circulation infarction: (1) blood brain barrier impairment in posterior circulation infarction as demonstrated by HARM can be observed with a frequency comparable to that reported in the medical literature, (2) HARM is associated with vessel recanalization, minor infarction growth as well as smaller infarction volumes on follow-up MRI in posterior circulation infarction, and (3) HARM in posterior circulation infarction can be detected up to 21 hours after intravenous gadolinium-based contrast agents application.

A possible pathomechanism for the changes of blood brain barrier permeability after acute ischemia is the activation of inflammatory processes and proteolytic enzymes [15]. The disruption of blood brain barrier may result in diapedesis of blood and consecutive hemorrhagic transformation [16]. Although HARM has initially been shown to be associated with larger ischemic lesion volumes, hemorrhagic transformation and poor clinical outcome [14], later studies did not find such an association [13,17]. Recently, Lee and co-workers reported a significantly lower frequency of blood brain barrier impairment in posterior circulation infarction as detected by permeability images derived from DSC PWI and a lower frequency of secondary intracerebral hemorrhage [5]. In the present study, blood brain barrier injury as demonstrated by HARM could be observed in half of the patients with PCA occlusion and in one third of the patients with BA occlusion, which is in accordance with earlier studies on HARM in acute ischemic stroke [7,13,14,18]. Consequently, it does not seem justified to assume fundamental differences in changes of blood brain barrier permeability between posterior and anterior circulation infarction. Interestingly, HARM has been detected remote from the acute ischemic lesions in all cases. In line with this observation, patients with HARM had significantly smaller ischemic lesion volumes on follow-up MRI in comparison to patients without HARM. This may indicate a different extent of blood brain barrier impairment as detected by different MRI techniques, especially since gadolinium contrast–enhanced FLAIR images are considered much more sensitive than T1-weighted images for detecting low concentrations of gadolinium-based contrast agents in the subarachnoid space [19,20]. However, a comparison of HARM on FLAIR images and permeability images derived from DSC PWI or DCE PWI has never been performed. Moreover, HARM occurs predominantly in the subarachnoid space whereas the question of a possible parenchymal enhancement on FLAIR images has not been answered conclusively [18,21].

In the present study, HARM in posterior circulation infarction has been detected up to 21 hours after initial MRI and intravenous gadolinium-based contrast agent application. On first glance, this might appear counterintuitive, in particular with regard to the used dosage of gadoteric acid and its half-life in plasma which is approximately 90 minutes. However, while it is well-known that it takes approximately 10 minutes to detect HARM on FLAIR images after intravenous gadolinium-based contrast agents application [7], systematic studies on how long HARM can be detected on FLAIR images thereafter have not been published yet. In a smaller case series of 11 patients, HARM has been detected in transient ischemic attack and stroke patients without a history of renal insufficiency within an interval of 1 day. In some patients with renal insufficiency the time interval was even higher (up to 6 days) [22]. In the present study, only two patients had a history of chronic kidney disease and in only one of these, HARM could be demonstrated on follow-up FLAIR images. However, in the respective patient the time from initial to follow-up MRI was the longest with 21 hours. Consequently, neuroradiologists should be aware of the phenomenon in patients with acute ischemic infarction who undergo short interval follow-up MRI after initial stroke MRI including DSC PWI, and even more in patients with chronic kidney disease.

The present study has some limitations. First, this is a retrospective clinical study of small size. However, to our knowledge this is the first series of patients investigating HARM in posterior circulation infarction in detail. Second, the time interval between contrast agent application and follow-up MRI was significantly longer in patients without HARM phenomenon. Thus, we cannot exclude that in some cases HARM might have been missed due to the longer interval between MRI examinations. Third, the study was performed with different MRI scanners and different imaging sequences. However, the MRI sequences, in particular the FLAIR and PWI sequences, have been customized for optimal comparability in daily clinical routine and consequently are generally comparable. Finally, the hospital-based retrospective study design might cause several types of bias and statistical errors such as selection bias, sample bias, or image-based selection bias.

In conclusion, HARM is a frequent finding in posterior circulation infarction and associated with vessel recanalization, minor infarction growth as well as smaller infarction volumes in the course. Radiologists and neuroradiologists should be cognizant of the fact that HARM may be present on short interval follow-up MRI in patients with acute ischemic infarction who initially underwent MRI and received intravenous gadolinium-based contrast agents.

## Supporting Information

S1 Table Detailed demographic characteristics, and MRI findings of patients included in the analysis. (XLSX)
